# Safety of Esophageal Cancer Surgery During the First Wave of the COVID-19 Pandemic in Europe: A Multicenter Study

**DOI:** 10.1245/s10434-021-09886-z

**Published:** 2021-04-08

**Authors:** Alexander B. J. Borgstein, Stefanie Brunner, Masaru Hayami, Johnny Moons, Hans Fuchs, Wietse J. Eshuis, Suzanne S. Gisbertz, Christiane J. Bruns, Philippe Nafteux, Magnus Nilsson, Wolfgang Schröder, Mark I. van Berge Henegouwen

**Affiliations:** 1 Department of Surgery, Amsterdam UMC, University of Amsterdam, Cancer Center Amsterdam, Amsterdam, The Netherlands; 2 Department of General, Visceral, Cancer and Transplantation Surgery, University Hospital of Cologne, Cologne, Germany; 3 Division of Surgery, Department of Clinical Science, Intervention and Technology (CLINTEC), Karolinskja Institutet, Solna, Sweden; 4 Department of Upper Abdominal Diseases, Karolinska University Hospital, Stockholm, Sweden; 5 Department of Surgery, Universitair Ziekenhuis Leuven, Leuven, Belgium

## Abstract

**Background:**

Many hospitals postponed elective surgical care during the first wave of the coronavirus disease 2019 (COVID-19) pandemic. Some centers continued elective surgery, including esophageal cancer surgery, with the use of preoperative screening methods; however, there is no evidence supporting the safety of this strategy as postoperative outcomes after esophageal cancer surgery during the COVID-19 pandemic have not yet been investigated.

**Methods:**

This multicenter study in four European tertiary esophageal cancer referral centers included consecutive adult patients undergoing elective esophageal cancer surgery from a prospectively maintained database in a COVID-19 pandemic cohort (1 March 2020–31 May 2020) and a control cohort (1 October 2019–29 February 2020). The primary outcome was the rate of respiratory failure requiring mechanical ventilation.

**Results:**

The COVID-19 cohort consisted of 139 patients, versus 168 patients in the control cohort. There was no difference in the rate of respiratory failure requiring mechanical ventilation (13.7% vs. 8.3%, *p* = 0.127) and number of pulmonary complications (32.4% vs. 29.9%, *p* = 0.646) between the COVID-19 cohort and the control cohort. Overall, postoperative morbidity and mortality rates were comparable between both cohorts. History taking and reverse transcription polymerase chain reaction (RT-PCR) were used as preoperative screening methods to detect a possible severe acute respiratory syndrome coronavirus 2 (SARS-CoV-2) infection in all centers. No patients were diagnosed with COVID-19 pre- or postoperatively.

**Conclusion:**

Esophageal cancer surgery during the first wave of the COVID-19 pandemic was not associated with an increase in pulmonary complications as no patients were diagnosed with COVID-19. Esophageal cancer surgery can be performed safely with the use of adequate preoperative SARS-CoV-2 screening methods.

**Supplementary Information:**

The online version contains supplementary material available at 10.1245/s10434-021-09886-z.

Esophageal cancer is the sixth leading cause of cancer-related deaths worldwide.^1^,^2^ Curative treatment for locally advanced esophageal cancer consists of esophagectomy combined with perioperative (radio-)chemotherapy.^3^,^4^ Esophagectomy is a complex surgical procedure and is associated with substantial morbidity, in particular postoperative pneumonia and consecutive respiratory failure.^5^–^8^

Many hospitals postponed elective surgical care during the first wave of the coronavirus disease 2019 (COVID-19) pandemic. This was necessary as medical resources were shifted to increase intensive care unit capacities, to prevent patients acquiring in-hospital severe acute respiratory syndrome coronavirus 2 (SARS-CoV-2) infections, and concerns regarding the safety of healthcare workers and patients.^9^,^10^ This strategy is supported by a recent study demonstrating that patients with a SARS-CoV-2 infection undergoing surgery have increased risk for postoperative pulmonary complications and mortality.^11^ Additionally, patients scheduled for esophageal cancer surgery are at high risk for symptomatic COVID-19 because of epidemiologic characteristics such as high age, male sex, and high prevalence of obesity; immunosuppression due to neoadjuvant therapy; high prevalence of pre-existing pulmonary comorbidities; and transthoracic esophagectomy with single lung ventilation.^12^–^14^

On the other hand, some countries have implemented national guidelines advising the use of preoperative SARS-CoV-2 screening methods in order to continue elective surgery.^15^ Certain international tertiary hospitals specializing in esophageal cancer have been able to continue elective cancer surgery with the use of preoperative screening; however, there is no evidence supporting the safety of this strategy as postoperative outcomes after esophageal cancer surgery during the COVID-19 pandemic have not yet been investigated in detail.

Currently, second waves of COVID-19 are occurring around the world; therefore, it is important to investigate the safety of continuing elective cancer surgery as postponement substantially increases the number of avoidable cancer deaths, as demonstrated in a recent national cancer registry analysis.^16^ The aim of this current study was to assess the safety of patients undergoing elective esophageal cancer surgery during the COVID-19 pandemic, focusing on respiratory failure as the most critical condition of SARS-CoV-2 infection.

## Methods

This international, retrospective, multicenter cohort study was conducted at four European tertiary referral hospitals in The Netherlands, Germany, Belgium, and Sweden, all specializing in esophageal cancer surgery. All participating centers continued elective esophageal cancer surgery during the first wave of the COVID-19 pandemic. Ethical approval was waived by the Amsterdam UMC review board because of the observational nature of the study; this decision was approved by the Institutional Review Board of each participating center.

### Study Population

**Fig. 1:**
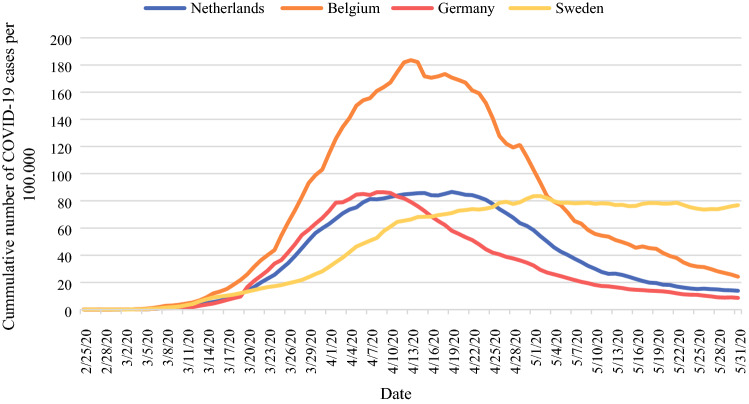
Daily number of new proven COVID-19 cases for the country of each participating center, calculated as the cumulative number for 14 days of COVID-19 cases per 100,000 population.^33^*COVID-19* coronavirus disease 2019

Consecutive adult patients undergoing elective esophageal cancer surgery were included in two cohorts. The first cohort consisted of patients who underwent esophagectomy between 1 October 2019 and 29 February 2020, i.e. the control cohort, while the second cohort consisted of patients operated between 1 March 2020 and 31 May 2020, i.e. the COVID-19 pandemic cohort. This study period reflects the months with the highest COVID-19 prevalence in the participating countries (Fig. 1).

Patients with the following characteristics were eligible for inclusion: age ≥18 years and undergoing either a thoracophrenicolaparotomy, transthoracic, or transhiatal esophagectomy, which could be performed as an open or totally minimally invasive procedure (including a hybrid procedure). Pre- and postoperative testing information for SARS-CoV-2 had to be available for patients in the COVID-19 pandemic cohort. Patients undergoing emergency esophagectomy were excluded. Patient data were only used if patients did not opt out for participation in this study.

### Severe Acute Respiratory Syndrome Coronavirus 2 (SARS-CoV-2) Testing

Each participating center provided information on the type of pre- and postoperative screening methods used in the COVID-19 cohort. In the case of a positive preoperative reverse transcription polymerase chain reaction (RT-PCR), surgery would be postponed for 2 weeks. A repeat RT-PCR test would be performed 2 days before the new date of surgery, although repeated PCR testing is known to be of limited value and is not advised in all national multidisciplinary guidelines.^17^ However, in the first wave, knowledge on COVID-19 was limited and repeat RT-PCR testing was performed because of the fear of in-hospital transmission.

### Data Collection

Patient demographics (age, sex, American Society of Anesthesiologists [ASA] score, and Charlson Comorbidity Index), tumor and treatment characteristics (histopathological staging, neoadjuvant therapy, type of surgery performed), and postoperative outcomes according to the Esophageal Complications Consensus Group (ECCG) definitions^18^ were collected from prospectively maintained databases in all centers. Additionally, pre- and postoperative screening results for SARS-CoV-2 infections (for patients in the COVID-19 pandemic group) were collected from the electronic patient record.

### Study Outcomes

The primary outcome was the rate of respiratory failure requiring mechanical ventilation in both cohorts, while secondary outcomes were overall postoperative morbidity, rate of postoperative pneumonia, number of postoperative SARS-CoV-2 infections, length of stay and hospital readmissions and mortality within 30 days postoperatively. The severity of postoperative complications was graded according to the Clavien–Dindo scale and the Comprehensive Complications Index (CCI).^19^,^20^

### Statistical Analysis

Univariate analyses of the two cohorts were compared using the Mann–Whitney U test for continuous variables, and the Chi-square or Fisher’s exact tests for categorial variables. To identify an association between undergoing esophageal cancer surgery during the COVID-19 pandemic and postoperative respiratory failure requiring mechanical ventilation, a multivariable logistic regression analysis was performed. All *p* values were based on two-sided tests and a *p* value < 0.05 was considered statistically significant. Data were analysed using SPSS version 26 (IBM Corporation, Armonk, NY, USA).

## Results

### Patient Characteristics

Between 1 March 2020 and 31 May 2020, a total of 139 patients underwent esophageal resection for cancer in the COVID-19 pandemic cohort. A total of 168 patients were included in the control cohort between 1 October 2019 and 29 February 2020. Baseline and treatment characteristics of all patients in both cohorts are presented in Table 1. Patients operated during the COVID-19 pandemic had a significantly higher ASA score. There were no differences in tumor and treatment characteristics between patients in both cohorts. Almost 75% of all patients in both cohorts underwent a minimally invasive esophagectomy.

**TABLE 1:** Baseline and treatment characteristics of all patients undergoing esophageal cancer surgery, as compared between the COVID-19 pandemic cohort (1 March 2020–31 May 2020) and the control cohort (1 October 2020–29 February 2020)

Characteristics	COVID-19 pandemic group [*N* = 139]	Control cohort [*N* = 168]	*p* value
Age, years [median (IQR)]	66 (58–71)	67 (60–73)	0.165
Male sex	116 (83.5)	141 (83.9)	0.911
BMI >25	49.3 (68)	100 (59.5)	0.073
ASA score			0.015
1	14 (10.1)	37 (22.0)	
2	81 (58.3)	90 (53.6)	
3	44 (31.7)	39 (23.2)	
4	0 (0.0)	2 (1.2)	
WHO performance status			0.431
0	75 (54.0)	86 (51.2)	
1	48 (34.5)	62 (36.9)	
2	12 (8.6)	15 (8.9)	
3	2 (1.4)	0 (0.0)	
4	1 (0.7)	0 (0.0)	
Missing	1 (0.7)	5 (3.0)	
Charlson comorbidity index			0.403
0	9 (6.5)	5 (3.0)	
1	24 (17.3)	26 (15.5)	
2	31 (22.3)	49 (29.2)	
3	36 (25.9)	29 (17.3)	
4+	39 (28.0)	59 (35.0)	
Comorbidities			0.254
Myocardial infarction	6 (4.3)	7 (4.2)	
Congestive heart failure	0 (0.0)	0 (00)	
Chronic pulmonary disease	14 (10.1)	14 (8.3)	
Diabetes mellitus (uncomplicated)	21 (15.1)	16 (9.5)	
Moderate to severe renal disease	0 (0.0)	2 (1.2)	
Multiple	4 (2.9)	12 (7.1)	
Histology			0.672
Adenocarcinoma	108 (77.7)	135 (80.4)	
Squamous cell carcinoma	28 (20.1)	30 (17.9)	
Other	3 (2.2)	2 (1.2)	
Clinical T stage			0.416
cT1	7 (5.0)	8 (4.8)	
cT2	13 (9.4)	23 (13.7)	
cT3	106 (76.3)	124 (73.8)	
cT4	3 (2.2)	10 (6.0)	
cTx	6 (4.3)	10 (6.0)	
Missing	4 (2.9)	0 (0.0)	
Clinical N stage			0.887
cN0	33 (23.7)	44 (26.2)	
cN1	30 (21.6)	33 (19.6)	
cN2	18 (12.9)	27 (16.1)	
cN3	3 (2.3)	4 (2.4)	
cNx	55 (39.6)	60 (35.7)	
Neoadjuvant therapy			0.958
Chemotherapy	36 (25.9)	46 (27.4)	
Chemoradiotherapy	77 (55.4)	91 (54.2)	
Surgical approach			0.280
Open	35 (25.2)	40 (23.8)	
Minimally invasive	104 (74.8)	125 (74.4)	
Minimally invasive converted to open	0 (0.0)	3 (1.8)	
Esophagectomy			0.705
Transhiatal	3 (2.2)	4 (2.4)	
Transthoracic	119 (85.6)	138 (82.1)	
Thoracophrenicolaparotomy	17 (12.2)	26 (15.5)	

Data are expressed as *n* (%) unless otherwise specified

*ASA* American Society of Anesthesiologists, *BMI* body mass index, *COVID-19* coronavirus disease 2019, *IQR* interquartile range

### Postoperative Outcomes

Table 2 shows the postoperative outcomes of all patients in both cohorts. There was no difference in the total number of postoperative complications (64.0% vs. 63.7%, *p* = 0.951), mean CCI score (44.3 vs. 39.7, *p* = 0.699), and maximum Clavien–Dindo score (*p* = 0.317).

**Table 2:** Postoperative outcomes of all patients undergoing esophageal cancer surgery, as compared between the COVID-19 pandemic cohort and the control cohort

	COVID-19 pandemic group [*N* = 139]	Control cohort [*N* = 168]	*p* value
Complications			
Yes	89 (64.0)	107 (63.7)	0.951
CCI score [mean (SD)]	41.2 (25.5)	39.8 (20.2)	0.699
Maximum Clavien–Dindo			0.317
I	5 (3.6)	8 (4.8)	
II	33 (23.7)	35 (20.8)	
III	25 (18.0)	39 (23.2)	
IV	25 (18.0)	24 (14.3)	
V	5 (3.6)	2 (1.2)	
Pulmonary complications	45 (32.4)	50 (29.9)	0.647
Pneumonia	20 (14.4)	32 (19.0)	0.297
Respiratory failure requiring reintubation	19 (13.7)	14 (8.3)	0.127
ICU admission	69 (49.6)	98 (58.3)	0.128
ICU admission, days [median (IQR)]	0 (0–4)	1 (0–3)	0.686
Length of hospital stay, days [median (IQR)]	12 (9–16.25)	12.5 (9–17.75)	0.430
Readmission within 30 days			
Yes	16 (11.5)	14 (8.3)	0.184
30-day mortality			
Yes	5 (3.6)	3 (1.8)	0.263

Data are expressed as *n* (%) unless otherwise specified

*CCI* Comprehensive Complications Index, *COVID-19* coronavirus disease 2019, *ICU* intensive care unit, *IQR* interquartile range, *SD* standard deviation

The percentage of respiratory failures requiring mechanical ventilation (13.7% vs. 8.3%, *p* = 0.127) and the total number of pulmonary complications were comparable between both cohorts (32.4% vs. 29.9%, *p* = 0.647). The ICU admission rate and length of stay at the ICU were similar in both cohorts. In one center, all patients were admitted to the ICU postoperatively as part of standard care (electronic supplementary Table S1). There was no difference in the 30-day readmission and mortality rates between the COVID-19 pandemic and control cohorts.

Electronic supplementary Table S1 provides an overview of the postoperative outcomes for each of the participating centers; no statistical differences between centers were observed.

In univariate logistic regression analysis, the odds ratio (OR) for postoperative respiratory failure requiring mechanical ventilation was 1.44 (95% confidence interval [CI] 0.80–2.58, *p* = 0.222) for patients operated during the COVID-19 pandemic. In a multivariate logistic regression, adjusted for ASA score, surgical approach and surgical procedure, the OR was 1.43 (95% CI 0.76–2.70, *p* = 0.272) for patients in the COVID-19 pandemic group.

### SARS-CoV-2 Testing Results

An overview of the screening methods used in each center is provided in electronic supplementary Table S2. All centers used COVID-19-specific symptom screening and RT-PCR; however, the date of implementation of the screening methods was different in each hospital.

SARS-CoV-2 testing results of patients in the COVID-19 pandemic cohort are presented in Table 3. Overall, 134/139 (96.4%) patients were screened for COVID-19 preoperatively and all were negative. History taking for specific COVID-19 symptoms was performed in most patients (95.0%), followed by white cell/lymphocyte count (73.4%), RT-PCR (71.9%), and chest computed tomography (CT; 11.5%). Thirty-six symptomatic patients (25.9%) received postoperative RT-PCR testing for SARS-CoV-2 and all patients tested negative. Overall, none of the patients were diagnosed with COVID-19, and subsequently no surgery was postponed because of screening results.

**Table 3:** Pre- and postoperative SARS-CoV-2 testing results of patients in the COVID-19 pandemic cohort

Preoperative	*N* = 139	Postoperative	*N* = 139
COVID-19		COVID-19	
Positive	0 (0.0)	Positive	(0.0)
Negative	134 (96.4)	Negative	36 (25.9)
Not tested	5 (3.6)	Not tested	103 (74.1)
Methods		Methods	
RT-PCR	100 (71.9)	RT-PCR	36 (25.9)
Chest CT	16 (11.5)		
Symptom screening	132 (95.0)		
White-cell/lymphocyte count	102 (73.4)		
Antibody analysis	0 (0.0)		
Surgery postponed			
No	139 (100.0)		

Data are expressed as *n* (%)

*COVID-19* coronavirus disease 2019, *RT-PCR* reverse transcription polymerase chain reaction, *CT* computed tomography, *SARS-CoV-2* severe acute respiratory syndrome coronavirus 2

## Discussion

This study investigated the safety of patients undergoing elective esophageal cancer surgery during the first wave of the COVID-19 pandemic in Europe and compared that with patients undergoing surgery in a period just before the COVID-19 pandemic. None of the patients in the COVID-19 pandemic cohort were pre- or postoperatively diagnosed with COVID-19. This resulted in a similar rate of patients with respiratory failure requiring mechanical ventilation in both cohorts. Therefore, undergoing esophagectomy during the COVID-19 pandemic was not associated with an increased risk of respiratory failure.

The ICU admission rate and length of stay at the ICU were comparable between both cohorts. In one center, all patients went to the ICU postoperatively as part of standard care. None of the participating centers experienced a shortage of ICU beds or delay in ICU readmission because of hospital COVID-19 volume during our inclusion period. The ASA score was higher in the COVID-19 pandemic cohort, with a higher percentage of patients with an ASA score of 2–3. There was no specific reason for this difference.

This is the first study to investigate the short-term postoperative outcomes in patients undergoing elective esophageal cancer surgery during the COVID-19 pandemic.

In the COVID-19 cohort, the percentage of patients with pulmonary complications (32.4%), the rate of respiratory failure requiring mechanical ventilation (13.7%), and the 30-day mortality rate (3.6%*)* were comparable with the findings of previous studies.^7^,^8^,^21^

A recent study by Chenchen et al. investigated the safety of performing cancer surgery during the COVID-19 pandemic.^22^ They found that none of the 621 patients tested positive for COVID-19 postoperatively. Shrikhande et al. performed a single-center prospective study examining 494 patients undergoing elective major cancer surgery in India and found that only six patients were diagnosed with COVID-19 postoperatively, none of whom required escalating care or intensive care treatment.^23^ In line with these findings, no patients were diagnosed with COVID-19 in our cohort, although the COVID-19 community prevalence in Europe was higher in our study period.

Studies performed at the beginning of the COVID-19 pandemic concluded that patients with a SARS-CoV-2 infection undergoing surgery had worse postoperative outcomes, with a high postoperative mortality rate.^24^ Increased 30-day mortality was associated with male sex, age >70 years, ASA score of 3–5, cancer surgery, and major surgical procedures. Additionally, oncologic patients undergoing surgery or chemotherapy have increased risk for severe COVID-19.^14^ Based on these findings, international societies advised to postpone elective surgery when possible, including esophageal cancer surgery.^13^ A study by the COVIDSurg group has estimated that the total percentage of adult elective operations that were cancelled during the first 12 weeks of the first wave of the COVID-19 pandemic was 72.3%.^25^ Globally, 37.7% of all cancer operations were cancelled or postponed. The study concluded that if countries increased their normal surgical capacity by 20% after the COVID-19 pandemic, it would take a median of 45 weeks to clear the accumulation of operations.^25^ The question remains whether postponement of cancer surgery leads to progression of the tumor and reduced overall survival. Turaga and Girotra investigated how long different types of cancer surgery could be safely delayed and concluded that most cancer surgeries can be safely delayed for at least 4 weeks without having a significant impact on patient survival or cancer progression.^26^ However, with a second COVID-19 wave currently developing in Europe, waiting lists will start to increase, which might lead to postponement of elective cancer surgery for more than 4 weeks.

A Dutch study evaluated the yield of preoperative screening for COVID-19 with chest CT and RT-PCR in asymptomatic patients. RT-PCR detected SARS-CoV-2 in at least 1 in every 100 asymptomatic patients undergoing elective or emergency surgery.^27^ This yield increased to 6% when the COVID-19 daily hospital admissions rate exceeded 1.5 per 100,000 inhabitants. The incremental yield of chest CT was only 0.4% and did not contribute to COVID-19 detection. None of the patients who underwent history taking and RT-PCR preoperatively developed symptomatic COVID-19 after surgery.^27^ In line with these findings, a recent study by the COVIDSurg group concluded that preoperative RT-PCR testing was beneficial before major surgery and in high SARS-CoV-2 risk areas.^28^ Having at least one negative preoperative RT-PCR test was associated with a lower rate of pulmonary complications (OR 0.68, 95 CI 0.68–0.98, *p* = 0.040).^28^

In our study, almost all patients in the COVID-19 cohort were screened for COVID-19-specific symptoms preoperatively and 70% underwent RT-PCR testing. RT-PCR testing was used as the standard preoperative screening method in all participating centers; however, because of limited testing capacity and differences in implications of national guidelines at the beginning of the COVID-19 pandemic, not all patients were tested with RT-PCR. Only symptomatic patients underwent RT-PCR testing for COVID-19 postoperatively.

Surgery would have been postponed for 2 weeks if a patient tested positive for COVID-19 preoperatively, with an additional RT-PCR test 2 days before the new date of surgery. This strategy would have been applied irrespective of whether a patient received neoadjuvant therapy. A previous study found that the interval between neoadjuvant therapy and esophagectomy could be safely extended to a maximum of 10 or more weeks.^29^

Seventy-five percent of all patients underwent minimally invasive surgery in both the COVID-19 pandemic cohort and the control cohort. According to the Society of American Gastrointestinal and Endoscopic Surgeons (SAGES) and the European Association of Endoscopic Surgery (EAES) guidelines, released during the first wave of the COVID-19 pandemic, minimally invasive surgery was discouraged as these procedures could contaminate surgical staff.^30^ Our study did not investigate the rate of SARS-CoV-2 infections among the surgical staff; however, as all patients were screened for COVID-19 preoperatively, the strategy of performing minimally invasive surgery in our cohort was safe.

Our study has some limitations. First, patients were included retrospectively from prospectively maintained databases in all centers; therefore, the participating centers did not use a similar standardized preoperative COVID-19 screening strategy during the inclusion period. Second, not all patients in the COVID-19 cohort were screened for COVID-19 preoperatively and 75% of patients were not tested postoperatively. Furthermore, only RT-PCR was used postoperatively to diagnose possible COVID-19 cases. RT-PCR is considered the reference standard to establish a SARS-CoV-2 infection, however sensitivity is considered to be moderate.^31^ Hence, asymptomatic COVID-19 patients or patients with false negative testing results could have been missed. However, clinical follow-up information was obtained for all patients and no patients were suspected for symptomatic COVID-19. Third, surgeons may have selected the healthiest patients to undergo surgery during the peak of the COVID-19 pandemic; therefore, this patient group might not be representative of the normal population undergoing esophageal cancer surgery. However, no differences were found in baseline characteristics between both cohorts.

We are currently facing a second COVID-19 wave in Europe, which is characterized by the appearance of new SARS-CoV-2 variants.^31^ Although it is unknown whether these new variants are more infectious, epidemiological data show that these variants have a higher transmissibility compared with the original variant.^31^,^32^ Hospitals will therefore face a higher number of COVID-19 patients during the upcoming months, which may affect the surgical and ICU capacity. Hence, hospitals that could continue elective cancer surgery during the first COVID-19 wave might have problems continuing cancer care during the second and possibly third waves. Increased lockdown measures and vaccination might prevent such a scenario.

## Conclusion

Elective esophageal cancer surgery can be performed safely during the COVID-19 pandemic with the use of adequate preoperative SARS-CoV-2 screening methods. With increasing numbers of operations being cancelled or postponed around the world, this study indicates that patients can undergo major cancer surgery during the ongoing COVID-19 pandemic without additional risk for the patient.

## Supplementary Information

Below is the link to the electronic supplementary material.

Supplementary file1 (DOCX 17 KB)
