# Supplementary material for: Safety of Esophageal Cancer Surgery During the First Wave of the COVID-19 Pandemic in Europe: A Multicenter Study
Source: Ann Surg Oncol. 2021 Apr 8;28(9):4805–13. doi: 10.1245/s10434-021-09886-z (PMC8028574; doi:10.1245/s10434-021-09886-z)
Supplement: Supplementary file 1 — Supplementary file1 (DOCX 17 KB) [file 10434_2021_9886_MOESM1_ESM.docx]

**Supplementary material**

**List of authors**

Alexander B.J. Borgstein, MD^1*^, Stefanie Brunner, MD^2*^, Masaru Hayami, MD, PhD^3^, Johnny Moons, MSc^4^, Hans Fuchs, MD, PhD^2^, Wietse J. Eshuis, MD, PhD^1^, Suzanne S. Gisbertz, MD, PhD^1^, Christiane J. Bruns, MD, PhD^2^, Philippe Nafteux, MD, PhD^4^, Magnus Nilsson, MD, PhD^3^, Wolfgang Schröder, MD, PhD^2#^, Mark I. van Berge Henegouwen, MD, PhD^1#^

^*^ Shared first authorship; these authors contributed equally to this article

^#^ Shared last authorship; these authors contributed equally to this article

**Affiliations**

1. Amsterdam UMC, University of Amsterdam, Department of Surgery, Cancer Center Amsterdam, the Netherlands
2. Department of General, Visceral, Cancer and Transplantation Surgery, University Hospital of Cologne, Cologne, Germany
3. Division of Surgery, Department of Clinical Science, Intervention and Technology (CLINTEC), Karolinskja Institutet and Department of Upper Abdominal Diseases, Karolinska University Hospital, Stockholm, Sweden
4. Department of Surgery Universitair Ziekenhuis Leuven, Leuven, Belgium

**Table S1.** Number of esophagectomies, surgical approach and postoperative outcomes in each of the participating centers.

|  | **Total (N=307)** | **Amsterdam UMC** | **University Hospital Cologne** | **UZ Leuven** | **Karolinska University Hospital** |
| --- | --- | --- | --- | --- | --- |
| Inclusions, no./total no. (%) |  | 58 (18.9) | 112 (36.5) | 107 (34.9) | 30 (9.8) |
| Surgical procedure, no./total no. (%) |  |  |  |  |  |
| Open | 75 (24.4) | 5 (8.6) | 16 (14.3) | 53 (49.5) | 1 (3.3) |
| Minimally invasive | 229 (74.6) | 53 (91.4) | 96 (85.7) | 53 (49.5) | 27 (90.0) |
| MI converted to open | 3 (1.0) | 0 (0.0) | 0 (0.0) | 1 (0.9) | 2 (6.7) |
| Type of resection, no./total no. (%) |  |  |  |  |  |
| Transthoracic | 257 (83.7) | 56 (96.6) | 111 (99.1) | 65 (60.7) | 25 (83.3) |
| Transhiatal | 7 (2.3) | 1 (1.7) | 1 (0.9) | 0 (0.0) | 5 (16.7) |
| Thoracophrenicolaparotomy | 43 (14.0) | 1 (1.7) | 0 (0.0) | 42 (39.3) | 0 (0.0) |
| Postoperative complications, no./total no. (%) |  |  |  |  |  |
| Yes | 196 (63.8) | 37 (63.8) | 63 (56.3) | 76 (71.0) | 20 (66.7) |
| Respiratory failure requiring mechanical ventilation | 33 (10.8) | 3 (5.3) | 11 (9.8) | 16 (15.0) | 3 (10.0) |
| Pneumonia | 52 (16.9) | 13 (22.4) | 12 (10.7) | 22 (20.6) | 5 (16.7) |
| ICU admissions | 167 (54.4) | 24 (41.4) | 111 (99.1) | 27 (25.2) | 5 (16.7) |
| 30-day mortality | 8 (2.6) | 1 (1.7) | 4 (3.6) | 3 (2.8) | 0 (0.0) |

**Table S2.** Preoperative SARS-CoV-2 screening methods used between 1 March, 2020 and 31 May, 2020 for patients undergoing esophageal cancer surgery in each of the participating centers.

| **Centers:** | **Screening methods:** | **Duration:** |
| --- | --- | --- |
| Amsterdam UMC | 1. Chest CT 2. Symptoms screening 3. RT-PCR | 1. 15 March – 23 April 2. 30 March – 31 May 3. 16 April – 31 May |
| Univeristy Hospital Cologne | 1. Symptoms screening 2. RT-PCR 3. White-cell / lymphocyte count | 1. 1 March – 31 May 2. 1 March – 31 May 3. 1 March – 31 May |
| UZ Leuven | 1. Symptoms screening 2. RT-PCR 3. White-cell / lymphocyte count | 1. 18 March – 31 May 2. 18 March – 31 May 3. 18 March – 31 May |
| Karolinska University Hospital | 1. Chest CT 2. Symptoms screening 3. RT-PCR | 1. 2 April – 31 May 2. 2 April – 31 May 3. 2 April – 31 May |
